# Anti‐fibrotic activity of *Euglena gracilis* and paramylon in a mouse model of non‐alcoholic steatohepatitis

**DOI:** 10.1002/fsn3.828

**Published:** 2018-11-08

**Authors:** Ayaka Nakashima, Ryota Sugimoto, Kengo Suzuki, Yuka Shirakata, Taishi Hashiguchi, Chikara Yoshida, Yoshihisa Nakano

**Affiliations:** ^1^ euglena Co., Ltd. Tokyo Japan; ^2^ SMC Laboratories, Inc. Tokyo Japan; ^3^ Center for Research and Development of Bioresources Osaka Prefecture University Osaka Japan

**Keywords:** *Euglena*, fibrosis, microalgae, NASH, Paramylon

## Abstract

Progression to non‐alcoholic steatohepatitis (NASH) manifests as hepatitis, fibrosis, and sometimes carcinoma, resulting in liver failure. Various clinical trials have indicated that several pharmacological agents, including angiotensin II receptor blockers (ARBs) or farnesoid X receptor (FXR) agonists, are effective in NASH treatment. In addition, functional foods are expected to be important alternatives for treating or preventing NASH. Recently, focus has been directed toward microalgae as dietary supplements, mainly for lifestyle‐related diseases, because they contain various nutrients and functional ingredients. Specifically, a unicellular microalga *Euglena gracilis* stores a unique β‐1,3‐glucan particle called paramylon that stimulates the immune system. In this study, we evaluated the effects of *Euglena* and paramylon on NASH in Stelic Animal Model (STAM) mice using Sirius red staining and confirmed that oral administration of *Euglena* or paramylon inhibits the process of liver fibrosis. Moreover, compared with controls, paramylon decreased non‐alcoholic fatty liver disease (NAFLD) activity scores related to inflammation. These results indicate that the oral administration of *Euglena* and paramylon inhibits fibrosis and ameliorates NASH.

## 1 INTRODUCTION

*Euglena gracilis* (*Euglena*) is a unicellular microalga that contains abundant nutrients, including vitamins and minerals, and has received much attention as a new ingredient of functional foods and feed (Aemiro et al., 2016; Matsumoto, Inui, Miyatake, Nakano, & Murakami, 2009). In addition, *Euglena* accumulates paramylon, a particulate β‐1,3‐glucan, as a carbon source. In a point of view of human health contributed by the β‐1,3‐glucan, it was reported that ingestion of barley or mushroom β‐glucans altered gut microbiota and mitigate many diseases (Friedman, 2016; Wang et al., 2016). So, oral administration of *Euglena* or paramylon can be considered beneficial for human health. Indeed, oral administration of *Euglena* has been shown to ameliorate colon cancer (Watanabe, Shimada, Matsuyama, & Yuasa, 2015) and type 2 diabetes (Shimada et al., 2016) in rodents. Similarly, the administration of paramylon improved atopic dermatitis (Sugiyama et al., 2010) and mitigated carbon tetrachloride‐induced liver injury (Sugiyama et al., 2009). Therefore, it is necessary to explore the functions of *Euglena* and paramylon further to obtain new insights into their potential as functional foods.

Non‐alcoholic steatohepatitis (NASH), a metabolic syndrome, is the extreme form of non‐alcoholic fatty liver disease (NAFLD) (Chalasani et al., 2012; Duvnjak et al., 2007). In most cases, NAFLD has few symptoms and is not accompanied by inflammation and fibrosis, whereas NASH may progress to cirrhosis and liver cancer without hepatitis virus infection or chronic alcohol intake. The incidence of NAFLD and NASH is rapidly increasing (Satapathy & Sanyal, 2015), and these diseases are expected to become a major global burden. Several studies have indicated that angiotensin II receptor blockers (ARBs) such as losartan and telmisartan or farnesoid X receptor (FXR) agonists may be effective in improving NASH symptoms (Arab, Karpen, Dawson, Arrese, & Trauner, 2016; Fujita et al., 2007; Hirata et al., 2013; Yoshiji et al., 2009); however, a definitive therapeutic option remains unavailable to date. Day et al. proposed a two‐hit theory for NASH development (Day & James, 1998), but it remains to be confirmed. Specifically, fatty liver is first induced by excessive nutrition that exceeds the liver storage limit, which in turn causes oxidative damage to liver cells, resulting in NASH development.

Many NASH models, including genetic models and/or dietary models, have been established thus far (Hebbard & George, 2011). Although methionine‐choline deficient (MCD) model is a typical diet‐induced model of NASH, it shows an opposite metabolic profile to that of human NASH. In contrast, in Stelic Animal Model (STAM) mice (Fujii et al., 2013; Saito et al., 2015), fatty liver is triggered by streptozotocin‐induced insulin resistance in combination with a high‐fat diet and may be observed in 6‐week‐old animals. Subsequently, steatohepatitis, fibrosis, and hepatocellular carcinoma appear by 8, 9, and 20 weeks, respectively. This pathological development shares similar characteristics to that of human NASH, suggesting that this model can be applied for the investigation of the effect of functional foods or drug candidates. In this study, we report the anti‐fibrotic effects of *Euglena* or paramylon in STAM mice.

## 2 MATERIALS AND METHODS

### 2.1 Test substance preparation

*Euglena* and paramylon were provided in powdered form by euglena Co., Ltd. (Tokyo, Japan). The components of *Euglena* have been described previously (Shimada et al., 2016). Briefly, *Euglena* is composed of 29.4% carbohydrates, 42.3% protein, and 19.0% fat. Approximately 70–80% of carbohydrates in *Euglena* were assumed to be paramylon. Paramylon isolation was conducted using a standard method (Inui, Miyatake, Nakano, & Kitaoka, 1982).

### 2.2 Animals and induction of NASH

**Figure 1:**
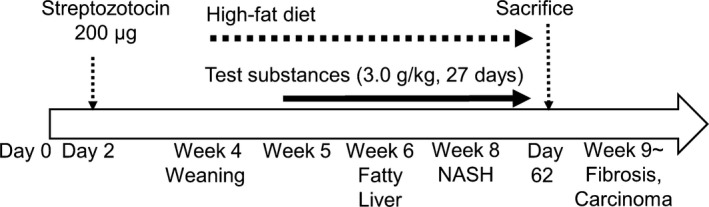
Experimental design and STAM mouse profile. (for details, see Materials and Methods)

Eighteen C57BL/6J SPF mice (14‐day pregnant females) were obtained from Japan SLC (Shizuoka, Japan). Male pups delivered naturally were selected for NASH induction. NASH was induced as previously described. Two‐day‐old male mice were subcutaneously injected with 200 μg streptozocin (20 μl/head) to confer insulin resistance. CE‐2 (CLEA Japan, Japan) was fed freely until the weaning period (4‐week‐old). After that, the high‐fat diet HFD32 (CLEA Japan, Japan) was fed to mice. Thirty‐six STAM mice were divided into six groups (six mice/group, three mice/cage) to equalize the average body weight by stratified random sampling on the day before initiation of test substance administration (Figure 1).

### 2.3 Administration of test substance

Saline solution was added to *Euglena* and paramylon dried powder just before administration.

In a single treatment, *Euglena*, paramylon (each 3.0 g/kg), and telmisartan (10 mg/kg) were orally administered with a feeding needle (10 ml/kg). Vehicle was also administered (10 ml/kg). Each treatment quantity (minimum adjustable quantity: 0.01 ml) was determined based on body weight on the day of treatment. Daily administration (once a day) started at 5 weeks of age and continued for 27 days.

### 2.4 Sirius red staining

Liver fibrosis was assessed with Sirius red staining. Sections from paraffin blocks were stained with 0.03% Sirius red solution (Waldeck, Germany) after hydrophilization. The stained sections were dehydrated for observation. The obtained pictures were analyzed using ImageJ software (National Institutes of Health).

### 2.5 Hematoxylin and eosin staining and NAFLD activity score calculation

Hematoxylin and eosin (HE) staining was performed as described previously (Fujii et al., 2013). NAFLD activity scores (NAS) were calculated based on the criteria of Kleiner et al. (Kleiner et al., 2005) (see also Table 1).

**Table 1:** Criteria for NAFLD activity scoring

Item	Score	Extent
Steatosis	0	<5%
	1	5%–33%
	2	>33%–66%
	3	>66%
Lobular inflammation	0	No foci
	1	<2 foci/200x
	2	2–4 foci/200x
	3	>4 foci/200x
Hepatocyte ballooning	0	None
	1	Few balloon cells
	2	Many cells/prominent ballooning

### 2.6 Immunohistochemistry of livers

For immunohistochemistry, sections were cut from frozen liver tissues embedded in Tissue‐Tek O.C.T. compound and fixed in acetone. Endogenous peroxidase activity was blocked with 0.03% H_2_O_2_ for 5 min, followed by incubation with Block Ace (Dainippon Sumitomo Pharma Co. Ltd., Osaka, Japan) for 10 min. The sections were incubated with a 100‐fold dilution of anti‐F4/80 antibody (BMA Biomedicals, Augst, Switzerland) at 4°C overnight. The sections were then incubated with biotin‐conjugated secondary antibody (VECTASTAIN Elite ABC kit, Vector Laboratories, Burlingame, CA, USA), followed by ABC reagent each for 30 min at 23~25°C. Enzyme‐substrate reactions were performed using 3, 3′‐diaminobenzidine/H_2_O_2_ solution (Nichirei Bioscience Inc., Tokyo, Japan). The sections were incubated with a 200‐fold dilution of anti‐α‐SMA antibody (rabbit monoclonal antibody, ab32575; Abcam, Cambridge, UK) at 4°C overnight. After incubation with secondary antibody (HRP‐goat anti‐rabbit antibody, Vector Laboratories, Inc.), enzyme‐substrate reactions were performed using 3, 3′‐diaminobenzidine/H_2_O_2_ solution (Nichirei Bioscience Inc.).

For quantitative analyses of inflammation and α‐SMA‐positive areas, bright field images of F4/80‐ and α‐SMA‐immunostained sections were captured around the central vein using a digital camera (DFC295; Leica, Wetzlar, Germany) at 200‐fold magnification. The positive areas in five fields/section were measured using ImageJ software (National Institute of Health, Bethesda, MD, USA).

### 2.7 RNA extraction and analysis of gene expression of liver injury and fibrogenic markers

Total RNA was extracted from STAM mice liver by using TRIzol reagent (Thermo Fisher Scientific, Waltham, MA, USA), according to the manufacturer's instructions. cDNA was synthesized from 50 ng of total RNA by using Rever Tra Ace qPCR RT Master Mix (Toyobo, Osaka, Japan). Real‐time quantitative polymerase chain reaction (RT‐qPCR) was performed in StepOne plus system (Thermo Fisher Scientific) using the PowerUP SYBR green Master mix (Applied Biosystems Inc., Warrington, UK). Target gene‐specific primers are listed in Table 2. The level of target gene expression was determined by comparing with a reference gene (*GAPDH*) based on 2^−ΔΔCt^ method (Livak & Schmittgen, 2001).

**Table 2:** Primers used in RT‐qPCR

Primers	Sequence (direction 5′ > 3′)	Reference
TNF‐α		
Forward	TCCAGCTGACTAAACATCCT	Oben et al. (2010)
Reverse	CCCTTCATCTTCCTCCTTAT	
IL‐6		
Forward	TTCACAGAGGATACCACTCC	Oben et al. (2010)
Reverse	GTTTGGTAGCATCCATCATT	
IL‐1β		
Forward	CTTTGAAGTTGACGGACCC	Mandrekar, Ambade, Lim, Szabo, and Catalano (2011)
Reverse	TGAGTGATACTGCCTGCCTG	
αSMA		
Forward	ATCTGGCACCACTCTTTCTA	Oben et al. (2010)
Reverse	GTACGTCCAGAGGCATAGAG	
Col1α2		
Forward	GAACGGTCCACGATTGCATG	Oben et al. (2010)
Reverse	GGCATGTTGCTAGGCACGAAG	
Col3α1		
Forward	CTGGTTTCTTCTCACCCTTC	This study
Reverse	GGCTTCCAGACATCTCTAGA	
MCP1		
Forward	CAGGTCCCTGTCATGCTTCT	This study
Reverse	GCTTCTTTGGGACACCTGCT	
GAPDH		
Forward	GTATGTCGTGGAGTCTACTG	This study
Reverse	GGTGCAGGATGCATTGCTGA	

### 2.8 Statistical analysis

Statistical analysis was performed using Bonferroni multiple comparison test or Student's *t *test using Prism 6 software (GraphPad Software, La Jolla, CA, USA). Comparisons were made between the following groups: (a) vehicle‐*Euglena*, (b) vehicle‐paramylon, and (c) vehicle‐telmisartan. The average of each group was expressed as means ± *SD*. *p* values < 0.05 were considered statistically significant. A trend or tendency was assumed when a Student's *t *test returned *p* values < 0.05.

## 3 RESULTS

### 3.1 General findings

**Figure 2:**
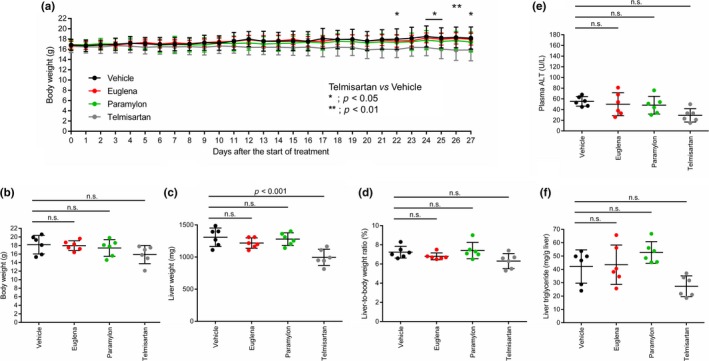
General findings in STAM mice administered test substances. Comparison of (a) body weight change, (b) body weight, (c) liver weight, (d) liver‐to‐body weight ratio, (e) plasma ALT, and (f) liver triglycerides in each group. Vehicle (black), Euglena (red), paramylon (green), telmisartan (gray). Asterisks indicate statistical significances (*p* < 0.01 or *p* < 0.05), while n.s. indicates “not significant”

To assess whether experimental design was appropriate or not, blood glucose levels were measured after *Euglena*, paramylon, and telmisartan treatment on STAM mice. As the results, the blood glucose level after *Euglena* and paramylon treatment was approximately same with the vehicle as control (average of blood glucose levels in each test group: vehicle; 670, Euglena; 640, and paramylon; 570 mg/dl). Furthermore, in case of telmisartan treatment, the average of blood glucose levels was 1070 mg/dl (Table 3). These results supported that the experimental design was appropriate, because the blood glucose level in each test group was same or higher than vehicle. The average body and liver weights of each group were subsequently measured. Telmisartan‐treated mice showed a significantly greater decrease in body weight than the vehicle group mice from days 24 to 27 (Figure 2a). A significant decrease of liver weight was also confirmed in addition to a decrease in body weight and liver‐body weight ratio (Figure 2b–d), suggesting that lipid accumulation was suppressed by telmisartan. In contrast, no significant changes were observed in *Euglena*, paramylon, and vehicle groups. Plasma alanine transaminase (ALT) and liver triglyceride levels are general biomarkers of liver condition and are suitable for monitoring NASH progression. Compared with the vehicle group, the telmisartan‐treated group showed a decrease in the levels of plasma ALT and liver triglycerides (Figure 2e,f). However, no differences were observed between the other groups.

**Table 3:** Plasma blood glucose

Plasma blood glucose(mg/dl)	Vehicle	Euglena	Paramylon	Telmisartan
Ave	668	644	567	1,072
*SD*	±103.6	±56.4	±61.6	±109.4

### 3.2 Sirius red staining area

**Figure 3:**
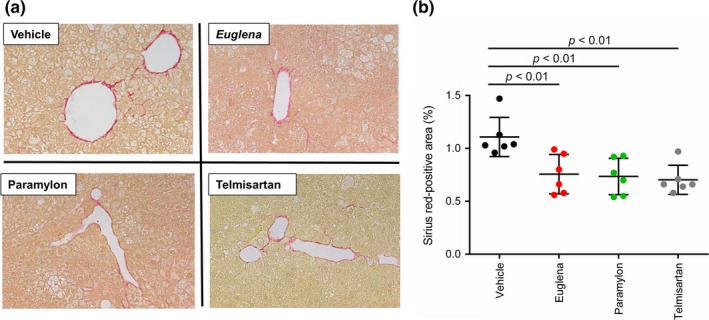
Sirius red staining of liver sections. (a) Photomicrographs of Sirius red staining from each treatment group, (b) comparison of Sirius red‐stained areas (*n* = 6) in each group; vehicle (black), Euglena (red), paramylon (green), telmisartan (gray). *p* < 0.01 indicate statistical significances

To evaluate liver damage due to NASH progression as indicated by collagen aggregation, Sirius red‐stained images were observed. In the vehicle group, positive staining reflecting collagen aggregation was clearly observed around the interlobular portal vein, sinusoid wall, and central vein wall (Figure 3a). Telmisartan, *Euglena*, and paramylon groups presented a significant decrease in positively stained area (average of positively stained area in each test group: vehicle; 1.11, Euglena; 0.76, and paramylon; 0.74, telmisartan; 0.70%) (Figure 3b). These results indicated that *Euglena* and paramylon treatment could contribute potentially to attenuate NASH progression on STAM mice.

### 3.3 NAFLD activity scores

**Figure 4:**
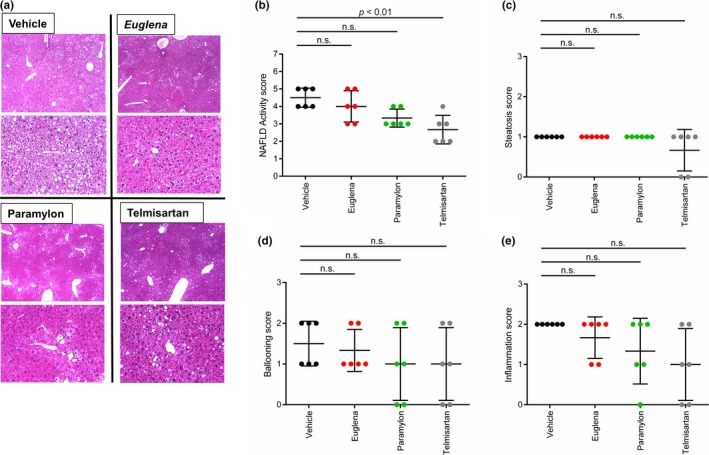
Non‐alcoholic fatty liver disease (NAFLD) activity scores. (a) Photomicrographs of HE‐stained liver sections in each treatment group (b) NAFLD activity scores; comparison of steatosis scores (c), ballooning (d) and inflammation scores (e). Vehicle (black), Euglena (red), paramylon (green), telmisartan (gray). n.s. indicates “not significant”

To confirm the attenuation effect of *Euglena* and paramylon on NASH progression, a histological assay was conducted against liver sections of each group by HE staining (see Materials and Methods section). As expected from the results of Sirius red staining assay (Figure 3), steatosis, lobular inflammation, and hepatocyte ballooning were observed in all groups (Figure 4a). Based on the criteria given in Table 1, NAS were calculated and compared between the groups. NAS of the telmisartan group were significantly lower than that of the vehicle group. Moreover, the paramylon group showed a decreasing trend of NAS (Figure 4b), although no change was observed in the *Euglena* group (average of NAS in each test group: vehicle; 4.5, Euglena; 4.0, and paramylon; 3.3, telmisartan; 2.7). Specifically, ballooning and inflammation scores, but not steatosis, contributed to the improvement of total score of the paramylon group (Figure 4c–e).

### 3.4 Immunostaining of liver

**Figure 5:**
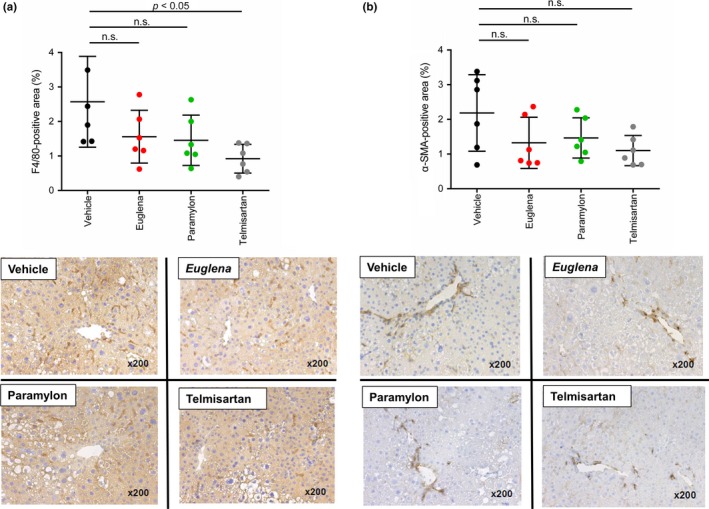
Immunostaining of liver (a) F4/80‐positive area (b) α‐SMA‐positive area. Vehicle (black), Euglena (red), paramylon (green), telmisartan (gray). *p* < 0.05 indicate statistical significances, while n.s. indicates “not significant”

To elucidate the mechanism underlying the anti‐NASH and anti‐fibrotic effects, immunostaining of F4/80, α‐SMA, and calculation of positive area ratio were performed using a liver sample. First, F4/80 reflecting the degree of inflammation was shown. In the telmisartan group, the positive area ratio due to F4/80 liver immunostaining was significantly decreased, while the *Euglena* and paramylon groups showed a decreasing trend in the positive area ratio due to F4/80 liver immunostaining (average of F4/80‐positive area in each test group: vehicle; 2.57, Euglena; 1.56, and paramylon; 1.46, telmisartan; 0.92%) (Figure 5a). Although no significant difference was observed, both *Euglena* and paramylon groups showed decreased positive area ratio of F4/80 by approximately 40% compared by vehicle. In contrast, α‐SMA reflecting the activation degree of hepatic stellate cells was shown. In the telmisartan, *Euglena,* and paramylon groups, the positive area rate due to immunostaining of α‐SMA liver showed a decreasing trend (average of α‐SMA‐positive area in each test group: vehicle; 2.19, Euglena; 1.32, and paramylon; 1.47, telmisartan; 1.10%) (Figure 5b). Although no significant difference was observed, both *Euglena* and paramylon groups showed decreased positive area ratio of α‐SMA by approximately 40% compared by vehicle.

### 3.5 Gene expression of liver injury and fibrogenic markers

**Figure 6:**
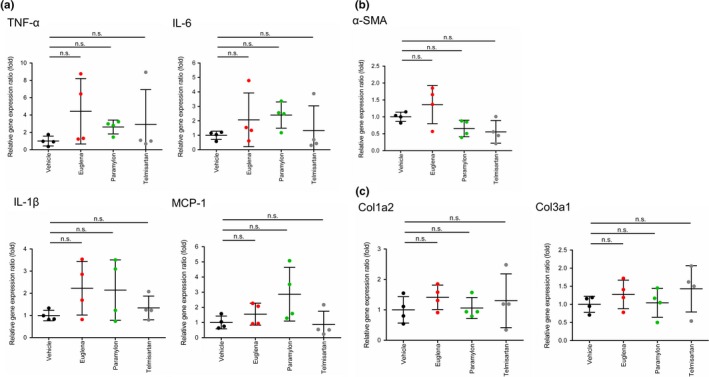
Gene expression of liver injury and fibrogenic markers (a) inflammation‐related genes (IL‐1β, IL‐6, TNF‐α, and MCP‐1) (b) α‐SMA gene expression. (c) collagen gene expression (Col1a2, Col3a1). Vehicle (black), Euglena (red), paramylon (green), telmisartan (gray). n.s. indicates “not significant”

Regarding inflammation‐related genes (IL‐1β, IL‐6, TNF‐α, and MCP‐1) in the *Euglena* and paramylon groups, the expression of each gene tended to increase compared with the control group. Regarding inflammation‐related genes in the telmisartan group, there was no increase in the expression level of each gene compared with the control group (Figure 6a).

In contrast, α‐SMA gene expression tended to decrease in telmisartan and paramylon groups (Figure 6b). Moreover, there was no clear difference in expression level of collagen gene (Col1a2, Col3a1; Figure 6c).

## 4 DISCUSSION

In this study, we evaluated the effect of *Euglena* and paramylon using STAM mice as an NASH model. Only the telmisartan group showed body and liver weight loss after 27 days, indicating the effectiveness of telmisartan as a pharmaceutical drug (Figure 1). Conversely, *Euglena‐* and paramylon‐treated groups presented amelioration of liver fibrosis; however, these groups did not show body and liver weight loss. Furthermore, the paramylon group showed a trend toward improved NAFLD score.

The progression of fibrosis is mainly attributed to inflammation and/or abnormal collagen accumulation (Caldwell & Argo, 2010; Friedman, 2010). The Sirius red positively stained area decreased in both *Euglena*‐ and paramylon‐treated groups, suggesting that collagen overproduction was suppressed by both test substances. In the paramylon group, NAFLD scores, especially inflammation scores, tended to decrease, suggesting that paramylon delayed fibrosis via inflammation inhibition. Regarding inflammation‐related genes, the expression levels in *Euglena*‐ and paramylon‐treated groups increased relative to the control group (Figure 6a). F4/80 immunostaining showed a trend toward decrease in positive area ratio (Figure 5a). Thus, gene expression and immunostaining showed opposite trends. It is suggested that this mismatch is caused by the time lag between gene and protein expression. Reduction in F4/80‐positive area ratio by these test substances is presumed to be due to the inhibition of swelling of positive cells and suppression of infiltration around positive vein. These test substances are activated by activation of liver macrophages/Kupffer cells. There is a possibility that macrophages/Kupffer cells may suppress the effect on migration.

Oxidative stress is linked to hepatocyte ballooning development, suggesting that radical scavengers act against NASH progression, as explained by the two‐hit theory (Day & James, 1998). Given that paramylon intake contributes to hepatoprotection through its antioxidant activity (Sugiyama et al., 2009), it is possible that this compound is also effective as a radical scavenger. Indeed, ballooning scores tended to improve in the paramylon group (Figure 4e). However, NAFLD scores did not improve in *Euglena*‐treated animals, suggesting that *Euglena* is not involved in the inhibition of inflammation, but may be effective in activating hepatic stellate cells, resulting in attenuation of collagen overproduction. A decrease in α‐SMA gene expression in the paramylon group and a decrease in α‐SMA‐positive area ratio of immunostaining in the *Euglena* and paramylon groups were observed, suggesting suppression of hepatic stellate cell activation (Figures 5b and 6b).

Since there was no clear difference in the expression level of collagen gene (Figure 6c), it was considered that *Euglena* and Paramylon may be involved in collagen metabolism and not collagen synthesis. In a previous epidemiological study, a strong association between diabetes and NAFLD was reported (Ortiz‐Lopez et al., 2012). It is known that *Euglena* inhibits hyperglycemia and bulimia in OLETF rats (Shimada et al., 2016), suggesting that this microalga may also affect insulin resistance and suppress fibrosis in STAM mice.

Blood directly reaches the liver via the portal vein from the gut, indicating that the liver is sensitive to the gut environment. It is already known that alteration of gut microbiota, as well as endotoxins produced by them, is related to NASH progression (Bashiardes, Shapiro, Rozin, Shibolet, & Elinav, 2016). The oral administration of *Euglena* provides multiple nutrients to the gut microorganisms of STAM mice. Therefore, we speculate that *Euglena* intake alters gut microbiota composition, which may lead to an anti‐fibrotic effect. Beta‐1,3‐glucan paramylon intake may also alter gut microbiota composition. However, utilization of paramylon as a carbon source for most microbiota seems to be difficult. Previously observed amelioration of autoimmune diseases, including atopic dermatitis (Sugiyama et al., 2010), supports that immune modulation is induced through gut microbiota alteration by *Euglena* or paramylon intake.

In this study, we used *Euglena* powder containing 20% paramylon. Hence, the *Euglena* group was administered paramylon as well. However, the anti‐inflammatory effect observed in the paramylon group was not observed in the *Euglena* group, indicating that the dosage of paramylon administered in the *Euglena* group was not sufficient for it to be effective. These results suggest that *Euglena* and paramylon inhibit fibrosis via different mechanisms. Hepatitis, fibrosis, and hepatocellular carcinoma are sequentially caused in fatty liver, resulting in liver failure. Therefore, it is inferred that the inhibition of fibrosis is the key in preventing carcinoma. *Euglena* and paramylon did not have as dramatic an effect as telmisartan did in the treatment of NASH. However, sustained intake of *Euglena* and paramylon as supplements may be effective for the prevention of NASH progression.

## ETHICAL STATEMENTS

This study was supported by euglena Co., Ltd. AN, OI, and KS are working in R&D department of euglena Co., Ltd. All animals were housed and cared for in accordance with the Japanese pharmacological Society Guidelines for Animal Use. All raw data are disclosed in Supporting Information Table S1.
